# Effect of the dietary supplementation with extracts of chestnut wood and grape pomace on performance and jejunum response in female and male broiler chickens at different ages

**DOI:** 10.1186/s40104-022-00736-w

**Published:** 2022-08-17

**Authors:** A. Pascual, M. Pauletto, A. Trocino, M. Birolo, M. Dacasto, M. Giantin, F. Bordignon, C. Ballarin, M. Bortoletti, G. Pillan, G. Xiccato

**Affiliations:** 1 Department of Agronomy, Food, Natural Resources, Animal and Environment (DAFNAE), University of Padova, Viale dell’Università 16, 35020 Padova, Legnaro Italy; 2 Department of Comparative Biomedicine and Food Science (BCA), University of Padova, Viale dell’Università 16, 35020 Padova, Legnaro Italy

**Keywords:** Growth, Immunohistochemistry, Meat quality, Tannins, Transcriptome

## Abstract

**Background:**

Recently, interest in the use of herbs and phytogenic compounds has grown because of their potential role in the production and health of livestock animals. Among these compounds, several tannins have been tested in poultry, but those from chestnut wood and grape-industry byproducts have attracted remarkable interest. Thus, the present study aimed to gain further insights into the mechanisms involved in the response to the dietary supplementation with extracts of chestnut wood or grape pomace. To this purpose, 864 broiler chickens were fed a control diet (C) or the same diet supplemented 0.2% chestnut wood (CN) extract or 0.2% grape pomace (GP) extract from hatching until commercial slaughtering (at 45 days of age) to assess their effects on performance, meat quality, jejunum immune response and whole-transcriptome profiling in both sexes at different ages (15 and 35 d).

**Results:**

Final live weight and daily weight gain significantly increased (*P* < 0.01) in chickens fed GP diets compared to CN and C diets. The villi height was lower in chickens fed the CN diet than in those fed the C diet (*P* < 0.001); moreover, a lower density of CD45^+^ cells was observed in chickens fed the CN diet (*P* < 0.05) compared to those fed the C and GP diets. Genes involved in either pro- or anti-inflammatory response pathways, and antimicrobial and antioxidant responses were affected by GP and CN diets. There was no effect of the dietary treatment on meat quality. Regarding sex, in addition to a lower growth performance, females showed a lower occurrence of wooden breast (16.7% vs. 55.6%; *P* < 0.001) and a higher occurrence of spaghetti meat (48.6% vs. 4.17%; *P* < 0.001) in *pectoralis major* muscles after slaughtering than those in males. Based on the results of whole-transcriptome profiling, a significant activation of some molecular pathways related to immunity was observed in males compared with those of females.

**Conclusions:**

The GP supplementation improved chicken performance and promoted immune responses in the intestinal mucosa; moreover, age and sex were associated with the most relevant transcriptional changes.

**Supplementary Information:**

The online version contains supplementary material available at 10.1186/s40104-022-00736-w.

## Background

During the last decade, interest in the use of herbs and phytogenic compounds has grown because of their potential role in the production and health of livestock animals. Among these compounds, plant tannins are polyphenolic compounds that exhibit antioxidant activity, which positively affect the composition of the gut microbial flora, stimulate the animal immune system and possess antibacterial properties [1–3]. Additionally, at the intestinal level polyphenols downregulate the expression of various proinflammatory cytokines, such as interleukin (IL)-1β, IL-4, IL-6, IL-10, tumor necrosis factor alpha (TNF-α), and interferon gamma [4].

In the poultry industry, several tannins have been tested, but those from chestnut wood (CN) and grape-industry byproducts have attracted remarkable interest. In vitro studies have shown the antimicrobial and antiparasitic effects of CN tannins [5, 6]. Moreover, in broiler chickens [7], CN extract increased jejunal mRNA levels of some growth-related antioxidant genes, such as epidermal growth factor and its receptor, and heat shock protein 70. However, results of in vivo trials lack consistency in their effects on poultry performance; positive effects have been reported by some authors [8, 9], whereas others [10, 11] did not find any improvement. Regarding byproducts of the grape-industry, supplementations with grape pomace (GP) concentrate and grape seed extracts have been shown to affect the gut morphology and increase the microflora biodiversity in broilers, in addition to improving growth performance [12].

Additionally, tannins from both CN and grape-industry byproducts exhibit antioxidant effects when supplemented in diets for broiler chickens [13, 14]. The antioxidant properties of tannins contained in GP have been observed in both the liver and breast meat of broiler chickens either after slaughter [14] or during storage [15]. Owing to their antioxidant properties, tannins can alleviate oxidative stress that occurs during the onset of breast myopathies [16, 17].

Interestingly, the effects of tannins could vary during animal development [10, 18], particularly with age and sex. Modern hybrids are highly precocious, and large sex-differences are observed from the very first days after hatching. Regarding age, animals fed a diet supplemented with a GP concentrate showed differences in performance and gut morphology at the end of an experimental trial, suggesting a time-dependent cumulative effect of tannins [19]. Humer et al. [20] reported remarkable differences in intestinal histometric and microbial traits between male and female broilers fed with phytogenic additives.

Thus, the present study aimed to gain further insights into the mechanisms involved in the response of broiler chickens of both sexes and different ages to different tannin extracts (i.e., CN and GP) supplemented in diets fed from hatching until slaughtering (45 days of age). In addition to chicken performance and meat quality, we examined the effect of dietary treatments on jejunum morphology, inflammatory patterns, and whole-transcriptome profiling.

## Methods

### Experimental facilities

This study was conducted at the Experimental Farm of the University of Padova (Legnaro, Padova, Italy) in a poultry house equipped with a cooling system, forced ventilation, radiant heating, and controlled light systems. Thirty-six wire-net pens (2.5 m × 2.4 m; 6 m^2^) were used, each equipped with five nipple drinkers and a circular feeder for manual distribution of feed. Each pen had a concrete floor covered with wood shaving litter (depth 5 cm, 2.5 kg/m^2^). A total of 24 h of light was provided during the first 2 d after the arrival of the chicks. Subsequently, the hours of light were progressively reduced until an 18L:6D photoperiod was achieved, which was then maintained from 13 days of age onward.

### Animals, experimental groups and in vivo recordings

A total of 864 broiler chicks (1 day old; 432 males and 432 females; Ross 308, Aviagen) were delivered by a commercial truck, in compliance with Council Regulation (EC) No 1/2005 to the experimental facilities. All chicks were vaccinated against Marek’s disease, infectious bronchitis, and Newcastle disease at the hatchery. They were randomly allocated in six experimental groups according to an experimental arrangement based on two sexes × three dietary treatments (control diet, CN diet, and GP diet), with 6 pens per experimental group and 24 birds per pen (total of 36 pens).

Chicks were individually weighed on the day of their arrival, identified by a plastic band at the leg, and weighed once per week to measure live weight and any difference among treatments at different ages, besides promptly identifying any health problem. The chickens were fed ad libitum. The pen feed consumption was measured daily through a computerized weighing system connected to all feeders.

The end of the growth trial was set at 44 d and birds were slaughtered at 45 days of age.

### Diets and feeding plans

Three commercial diets were formulated as the control (C) diets to be fed during three periods, i.e., from arrival to 15 d; from 15 to 29 d; and from 29 d until slaughtering (Table 1). These C diets, produced in a mash form, were supplemented with 0.2% CN extracts (CN diets) or 0.2% GP extract (GP diets). The CN extract (Saviotan® Feed, 57 mg/g phenolic compounds, GAE equivalent Folin-Ciocalteu) was produced by Sedepan (Radicofani, SI, Italy). The GP extract (77 mg/g phenolic compounds, GAE equivalent Folin-Ciocalteu) was produced by Tampieri Financial Group (Faenza, RA, Italy).

**Table 1:** Ingredients and chemical composition measured in the lab (if not specified otherwise) of the control (C) diets. These diets were supplemented with 0.2% chestnut wood (CN) extracts or 0.2% grape pomace (GP) extracts to obtain the CN and GP diets, respectively

Period of administration	1-15 d	15-29 d	29 d to slaughtering
Ingredients			
Corn meal, %	56.60	59.25	63.15
Soybean meal (CP 48%), %	34.50	30.00	24.60
Toasted full-fat soybean meal, %	3.00	5.00	7.00
Animal fat, %	2.50	2.50	2.50
Dicalcium phosphate, %	1.00	0.50	0.25
Calcium carbonate, %	0.92	1.33	1.30
Sodium chloride, %	0.26	0.27	0.27
L-lysine base, liquid (50%), %	0.25	0.24	0.16
Methionine hydroxy analogue, %	0.31	0.28	0.23
Vitamin-mineral premix^a^, %	0.30	0.30	0.30
6-phytase (EC 3.1.3.26), %	0.20	0.20	0.20
L-Threonine, %	0.11	0.08	0.04
Coccidiostat^b^, %	0.05	0.05	0.00
Chemical composition			
Dry matter, %	89.2	89.2	89.3
Crude protein, %	22.2	20.8	20.2
Ether extract, %	5.4	5.5	6.4
Crude fiber, %	1.3	1.2	1.8
Ash, %	5.7	5.5	6.6
Starch, %	41.1	43.3	40.2
Calcium^c^, %	0.81	0.80	0.71
Phosphorous^c^, %	0.58	0.47	0.42
Digestible phosphorous^c^, %	0.34	0.24	0.18
Digestible lysine^c^, %	1.32	1.23	1.05
Digestible methionine + cysteine^c^, %	0.91	0.85	0.76
Digestible threonine^c^, %	0.83	0.76	0.66
Apparent metabolizable energy^c^, kcal/kg	2982	3045	3087

^a^Premix provided per kg of feed: vit. A, 10,000 IU; vit. D_3_, 3500 IU; vit. E acetate, 90 mg; vit. K_3_, 6 mg; Biotin, 0.38 mg; Thiamine, 3.75 mg; Riboflavin, 8 mg; vit. B_6_, 5.75 mg; vit. B_12_, 0.1 mg; Niacin, 70 mg; Pantothenic acid, 17.5 mg; Folic acid, 2.25 mg; Fe, 45 mg; Cu, 10 mg; Mn, 70 mg; Zn, 65 mg; Se, 0.25 mg

^b^Sodium Monensin, 100 mg/kg feed

^c^Values calculated according to FEDNA [21]

The supplementation level of extracts was chosen based on commercial standards and previous results [8]. The inclusion of CN and GP extracts in the diets was performed at the experimental farm by thoroughly mixing the C diets with the dried extracts using an electric concrete mixer (Suncoo 4/5HP Concrete mixer, 140 L, 600 W, 2800 r/min; SUNCOO, China). A total of 3 kg of mash diet were progressively added with extracts in two steps (starting with 1 kg) and mixed by hand in a box prior mixing them with other 47 kg of mash diet in the electric concrete mixer.

Diets were analyzed to determine their dry matter content, crude protein, and crude fiber using AOAC [22] methods. The ether extract was analyzed after acid hydrolysis [23].

### Sampling of jejunum tissues

At 15 and 35 days post hatching, 36 chickens per period (1 chick per pen with mean BW) were selected and euthanized with CO_2_ asphyxiation, prior to jejunum tissue collection. One sample of approximately 2 cm was taken from the jejunum, at the midpoint between the end of the duodenal loop and the location of the Meckel’s diverticulum [24], and washed in phosphate-buffered saline (PBS). Sections (approximately 1-cm thick) were fixed in paraformaldehyde in PBS (0.1 mol/L, pH 7.4), dehydrated, embedded in paraffin at the laboratory, and later submitted to the histological analyses and immunohistochemistry as detailed in the following section.

Immediately before fixing, small jejunum sections were collected under RNase-free conditions and stored in RNAlater® reagent (Applied Biosystems, Foster City, CA, USA) for transcriptomic analyses. In the laboratory, RNA-seq samples were stored at 4 °C overnight and then transferred to −80 ºC until further processing.

### Histological analyses and immunohistochemistry

Two serial 4-μm sections per jejunum sample were obtained using a microtome and stained with hematoxylin/eosin for morphometric evaluation and Alcian blue (pH 2.5)-PAS method for quantitative analysis of goblet cells. Moreover, two further serial sections were used for CD3^+^ and CD45^+^ immunohistochemical analyses. The villi length and crypt depth were collected by a slide scanner (D-Sight, A. Menarini Diagnostics, Firenze, Italy) and measured using image analysis software (DP-soft, Olympus Optical, Co., Hamburg, Germany), according to the procedure described by Hampson [25]. The goblet cells positive for Alcian blue (pH 2.5)-PAS staining were counted using NIH ImageJ software [26] on 10 different villi per animal along 300 μm of the villus surface. Immunohistochemical analyses to identify CD3^+^ intraepithelial T-cells and CD45^+^ intraepithelial leukocytes in broiler jejunal mucosa were performed following the procedure described by Röhe et al. [27]. Intraepithelial leukocytes were counted in the epithelium using a reference rectangle with the short side at 100 μm and expressed as the density of CD45^+^ and CD3^+^ cells (cells/10,000 μm^2^).

### RNA-seq library preparation and sequencing

Total RNA from the 72 chickens slaughtered at 15 and 35 d was extracted using the RNAeasy Mini Kit (Qiagen, Hilden, Germany), following the manufacturer’s instructions; total RNA concentration was then determined using a Qubit RNA BR (Broad-Range) kit in a Qubit 2.0 Fluorometer (Life Technologies, Carlsbad, CA, USA). RNA quality was assessed using a 2100 Bioanalyzer (Agilent Technologies, Waldbronn, Germany). All samples had an RNA integrity number > 7.

Equal amounts of RNA from three different chickens of the same sex, fed with the same diet and slaughtered at the same age (15 or 35 d) were pooled; a total of 24 RNA pools were obtained (i.e., two pools/replicates per sex for each of the three diets and per two slaughtering age). Notably, the RNA pooling is a common practice among gene expression studies, and it is well justified based on statistical and practical considerations [28]. Twenty-four tagged RNA-seq libraries were prepared using the Illumina TruSeq Stranded mRNA kit and sequenced on an Illumina NovaSeq 6000 instrument at the NGS Sequencing Core (Padova, Italy) following a 100-bp paired-end approach.

### RNA-seq reads processing and mapping

Initial quality control was performed using FastQC software version 0.11.9 [29]. Read trimming and adapter removal were performed using Trimmomatic (version 0.39) with default parameters [30]. Reads shorter than 36 bp were excluded from the analysis. Residual ribosomal RNAs (rRNAs) were removed through the local sequence alignment tool SortMeRNA 2.1 [31] against different databases (Rfam 5.8S; Rfam 5S; Silva 16S archaeal, bacterial; Silva 18S eukaryote; Silva 23S archaeal, bacterial; Silva 28S eukaryote). Reads trimmed and cleaned as described above were then mapped against the chicken Ensembl reference genome (GCA_000002315.5) using the STAR aligner and following the two-pass mapping mode [32]. The maximum number of allowed mismatches and the maximum number of loci to which the reads could map were set to 8 and 10, respectively. Read counts per sample at the gene level were extracted by setting the GeneCounts quantification while running STAR.

### Commercial slaughtering and carcass and meat quality recordings

At 45 days of age, all remaining chickens were slaughtered in a commercial slaughterhouse. The chickens were weighed individually before crating. Loading took approximately 1 h, transport from the experimental facilities to the commercial slaughterhouse took approximately 15 min, and lairage before slaughtering took approximately 3 h. Ready-to-cook carcasses were recovered after 2 h of refrigeration at 2°C and individually weighed to measure the slaughter dressing percentage.

A total of 144 carcasses (four per pen), previously selected on the basis of the final live weight as corresponding to the mean BW within a pen, were subjected to gross examination to evaluate the occurrence in *pectoralis major* muscles (presence or absence) of white striping (WS) (either moderate or severe) [33]; wooden breast (WB) (firm upon palpation, prominent ridge like bulge on caudal area of fillet, clear viscous fluid cover and/or petechial multifocal lesions on the fillet surface) [34]; and spaghetti meat (SM) (exhibiting an overall impaired integrity and tendency toward separation of the muscle fiber bundles especially within the cranial part of the fillet) [35]. The 144 carcasses were stored at 2 °C before meat quality analyses. Twenty-four hours after slaughter, carcasses were dissected for the main cuts (breast, wings, thighs, and drumsticks). *Pectoralis major* muscles were separated from the breasts for meat quality analyses [36]. The pH values of the *pectoralis major* muscles were measured in triplicates on their ventral side with a pH meter (Basic 20, Crison Instruments Sa, Carpi, Italy) equipped with a specific electrode (cat. 5232, Crison Instruments Sa, Carpi, Italy). The L*a*b* color indexes were measured in triplicate on the ventral side of the same muscles covered by a transparent plastic film, using a Minolta CM-508 C spectrophotometer (Minolta Corp., Ramsey, NJ, USA) [37].

After measuring the pH and color indexes, one meat portion (8 cm × 4 cm × 3 cm) was separated from the cranial side of the *pectoralis major* muscle, parallel to the direction of the muscle fibers, and stored under vacuum in plastic bags at -18 °C until meat analyses. Thawing and cooking losses were measured in this cut [36]. After thawing, the meat portion was placed in a plastic bag and cooked in a water bath for 45 min until an internal temperature of 80 °C was achieved. After 40 min of cooling, another meat portion (4 cm × 2 cm × 1 cm) was separated to assess the maximum shear force using an LS5 dynamometer (Lloyd Instruments Ltd, Bognor Regis, UK) using the Allo-Kramer (10 blades) probe (load cell: 500 kg; distance between the blades: 5 mm; thickness: 2 mm; cutting speed: 250 mm/min) [37].

The *pectoralis major* muscles of the remaining 72 carcasses (2 chickens per pen, 12 per experimental group) were dissected and subsequently stored at -20 °C to assess the meat oxidation level of thiobarbituric acid reactive substances (TBARs) [38] using spectrophotometric measurements (Jasco Mod. 7800 UV/VIS) at 532 nm. The results were expressed as μg of malondialdehyde/kg.

### Statistical analysis

Individual data of live weights, daily growth, and carcass and meat traits were subjected to analysis of variance (ANOVA) with diets (C, CN, and GP) and sex as main factors of variability and their interactions, and the pens as a random effect, using the PROC MIXED procedure in SAS [39]. Being live weight of male and female chicks significantly different at the first hatching day (Table 2) it was included in the model as a covariate for live weights and daily growth. Pen data of feed intake and feed conversion were subjected to ANOVA, with diet and sex as main factors of variability, and their interactions, using the PROC GLM procedure [39]. Individual data related to jejunum morphology, goblet cells, and CD3^+^ and CD45^+^ cell densities were analyzed using the PROC GLM procedure, with diet, age, and sex as the main effects and their interactions. The Chi-square test was used to test differences in mortality according to diet and the rate of myopathies according to diet and sex. Adjusted means were compared using Bonferroni’s *t-*test. Differences between the means with *P* ≤ 0.05 were considered statistically significant.

For whole-transcriptome profiling, a pairwise differential expression (DE) analysis was performed using the likelihood ratio test implemented in EdgeR [40] to compare mRNA profiles between experimental groups. A false discovery rate (FDR) of ≤ 0.05 and a fold change (FC) of ≥ 1.5 were used as thresholds of significance between age and sex. Regarding changes due to diets, an FC threshold of 2 was selected to mitigate possible false positives due to the limited number of replicates per experimental group (i.e., two per diet, per age, per sex). Overall, four sex/age-specific datasets (each consisting of six samples) were analyzed separately, i.e., females at 15 d; females at 35 d; males at 15 d; and males at 35 d. A functional interpretation of significant differentially expressed genes (DEGs) was obtained through a Gene Ontology over-representation test, performed using the ClusterProfiler package in the R environment [41]. Only KEGG pathways were considered, using the function enrichKEGG. Ensembl gene identifiers were used to establish a list of significantly upregulated and downregulated genes and a “background” (i.e., the whole set of expressed genes). The ClusterProfiler package was used to produce plots representing enriched terms (*P* ≤ 0.05).

A pre-ranked KEGG Gene Set Enrichment Analysis (GSEA) [42] was performed to investigate whether gene sets defined *a priori* showed a statistically significant enrichment at either end of the ranking. A statistically significant enrichment value (Benjamini–Hochberg adjusted *P* value ≤ 0.05) indicates that the biological activity (e.g., the biomolecular pathway) characterized by the gene set is correlated with the supplied ranking. The input was prepared as follows: the raw *P* values (pval) obtained through the pairwise DE analysis were used to rank the list of genes by significance. When multiple genes with the same gene name were detected, only the most significant gene (based on pval) was retained. The pval were replaced by 1-pval or -(1-pval) when a gene was over- or under-expressed, respectively. The analysis was performed using the gseKEGG function in the ClusterProfiler package [41].

## Results

### Growth performance

Live weight at 15 d did not differ among groups (513 g on average), whereas live weight at 29 d was higher in broilers fed the GP diet than in those fed the CN diets (*P* < 0.01), and this difference was confirmed at the end of the study in comparison with those fed the C and CN diets (45 d; *P* < 0.01) Table 2. Therefore, the daily weight gain during the study was higher in broilers fed the GP diet than in those fed the other two diets (*P* < 0.01), without differences in feed intake and feed conversion.

**Table 2:** Growth performance^1^ (LS means) and mortality of broiler chickens until slaughter

	Diet (D)			Sex (S)			*P* value		MSE
Items	C	CN	GP	Females	Males	D	S	D×S	
Chickens, n	258	252	255	383	382				
Pens, n	12	12	12	18	18				
Live weight, g									
Initial (1 d)	44.3	43.9	44.5	43.7	44.9	0.15	<0.001	0.16	3.45
15 d	512	510	518	498	529	0.23	<0.001	0.12	50.7
29 d	1689^ab^	1669^a^	1703^b^	1559	1815	<0.01	<0.001	0.12	124
Final (44 d)^2^	3099^a^	3087^a^	3146^b^	2816	3406	<0.01	<0.001	0.02	217
Whole trial (1-44 d)									
Daily weight gain^2^, g/d	69.4^a^	69.1^a^	70.5^b^	63.0	76.4	<0.01	<0.001	0.02	4.94
Daily feed intake, g/d	111	110	112	102	119	0.16	<0.001	0.70	2.47
Feed conversion	1.59	1.59	1.59	1.59	1.59	0.85	0.91	0.26	0.36
Losses^3^, %	2.27	4.55	3.41	3.28	3.54	0.33	0.70	0.70	-

*MSE* root mean square error. *C*, control diet. *CN*, control diet supplemented with 0.2% chestnut wood extracts. *GP*, control diet supplemented with 0.2% grape pomace extracts

^1^Individual data: live weight and daily growth rate. Pen data: feed intake and feed conversion

^2^Interaction Diet × Sex, Final live weight: *P* = 0.02: 2827 g, 2789 g and 2822 g in females fed C, CN and GP diets; 3372 g, 3381 g and 3473 g in males fed C, CN, and GP diets, respectively. Daily weight gain: *P* = 0.02: 63.2 g/d, 62.4 g/d and 63.1 g/d in females fed C, CN, and GP diets, 75.6 g/d, 75.8 g/d and 77.9 g/d in males fed C, CN, GP diets, respectively.

^3^Dead and lame chickens

^a,b^Values with different superscript letters significantly differ *(P* < 0.05)

Males were heavier than females from the first day to the end of the study (*P* < 0.001), resulting in a higher daily weight gain (+22%) and feed intake (+17%), without differences in feed conversion (1.59) (Table 2). A significant interaction was observed between dietary treatment and sex at 45 d (*P* < 0.05); males fed the GP diet were heavier than those fed C and CN diets, whereas females fed C and GP diets were heavier than those fed the CN diet.

Losses were low (3.4%, 27 chickens) and due to mortality (1.1%) and lameness (2.3%) (Table 2).

### Slaughter results, meat quality, and myopathy rate

Table 3 shows that the carcass weight (with feet) was higher in chickens fed the GP diet than in those fed the other two diets (*P <* 0.05). No diet-related effects on myopathy occurrence (Table 3) or meat quality (Table 4) were recorded.

**Table 3:** Slaughter results, carcass traits (LS means) and myopathy rates in chickens slaughtered at 45 days of age

Items	Diet (D)			Sex (S)		*P *value			MSE
	C	CN	GP	Females	Males	D	S	D×S	
Chickens^1^, n	258	252	255	383	382				
Pens, n	12	12	12	18	18				
Cold carcasses^2^, g	2352^a^	2342^a^	2392^b^	2122	2602	0.03	<0.001	<0.01	173.06
Dressing out percentage, %	76.6	76.8	76.8	76.4	77.1	0.45	<0.001	0.51	1.48
Chickens, n	48	48	48	72	72				
Cold carcasses^3^ (CC), g	2274	2243	2306	2063	2486	0.32	<0.001	0.10	143.58
Dressing percentage, %	73.3	73.0	73.0	73.3	73.0	0.48	0.18	0.92	1.32
Breast yield^4^, % CC	39.3	38.8	39.5	40.0	38.3	0.16	<0.001	0.56	1.76
*P. major*, % CC	12.2	12.1	13.4	12.5	11.9	0.33	<0.001	0.37	0.85
Wings, % CC	9.8	10.0	9.9	9.9	9.9	0.06	0.44	0.65	0.51
Legs (thighs+drumsticks), % CC	30.1	30.1	29.8	29.6	30.3	0.67	0.04	0.43	2.02
Myopathy rates at *P. major*									
White striping, %	72.9	60.4	79.2	66.7	75.0	0.12	0.26	-	-
Wooden breast, %	41.7	25.0	41.7	16.7	55.6	0.11	<0.001	-	-
Spaghetti meat, %	29.5	25.5	25.0	48.6	4.2	0.83	<0.001	-	-

*MSE* root mean square error. *C*, control diet. *CN*, control diet supplemented with 0.2% chestnut wood extracts. *GP*, control diet supplemented with 0.2% grape pomace extracts

^1^Carcasses with feet. ^2^Interaction Diet × Sex, Cold carcasses: *P* < 0.01: 2126 g, 2103 g, 2126 g in females fed C, CN and GP diets, respectively; 2571 g, 2580 g, and 2652 g in males fed CN and GP diets. ^3^Without feet. ^4^With bone

^a,b^Values with different superscript letters significantly differ *(P* < 0.05)

**Table 4:** Rheological traits and lipid oxidation status (TBARs) of the pectoralis major muscle in chickens slaughtered at 45 days of age

Items	Diet (D)			Sex (S)		*P-*value			MSE
	C	CN	GP	Females	Males	D	S	D×S	
*P. major*, n	48	48	48	72	72				
pH	5.99	5.99	5.95	5.94	6.02	0.56	<0.01	0.99	0.16
L*	50.4	50.2	49.7	49.9	50.1	0.66	0.63	0.10	2.40
a*	-0.04	-0.07	-0.15	-0.03	-0.15	0.56	0.23	0.20	0.54
b*	10.4	10.3	10.2	10.5	10.1	0.70	0.09	0.65	1.38
*P. major*, n	24	24	24	36	36				
Cooking losses, %	12.0	11.7	11.8	11.4	12.3	0.99	0.65	0.92	12.4
Shear force, kg/g	4.17	4.06	4.30	4.17	4.18	0.39	0.96	0.72	0.83
TBARs, mg MDA/kg	0.083	0.080	0.075	0.078	0.080	0.38	0.74	0.22	0.021

*MSE* root mean square error. *C*, control diet. *CN*, control diet supplemented with 0.2% chestnut wood extracts. *GP*, control diet supplemented with 0.2% grape pomace extracts

Regarding the effect of sex, males had heavier carcasses (+17%, *P <* 0.001) and a higher proportion of leg weight (thighs + drumsticks) (+0.7; *P* < 0.05) but a lower breast yield (-1.7%; *P* < 0.001) and *pectoralis major* muscle proportion (-0.6%; *P* < 0.001) than those of females (Table 3). Females showed a lower meat pH (*P* < 0.01) (Table 4), lower WB rate (*P* < 0.001), and higher SM rate (*P* < 0.001) than those of males but had similar WS rates to those of males (Table 3).

### Jejunum morphology and immuno-histochemical analyses

On average of the two slaughtering times of 15 and 35 d, the villi height was lower in chickens fed the CN diet than in those fed the C diet (*P* < 0.001); moreover, chickens fed the CN diet had a lower density of CD45^+^ cells (*P* < 0.05) than those chickens fed the GP diet (Table 5). Dietary treatment did not affect the density of goblet cells.

**Table 5:** Jejunum morphometry, number of goblet cells and densities of CD45^+^ and CD3^+^ cells at 15 and 35 days of age

Items	Diet (D)			Age (A)		Sex (S)		*P-*value							MSE
	C	CN	GP	15	35	F	M	D	A	S	D×A	A×S^1^	D×S^2^	D×A×S	
Broilers, n	24	24	24	36	36	36	36								
Villi height, μm	1033^b^	934^a^	954^ab^	866	1082	936	1011	<0.001	<0.01	<0.01	0.44	0.02	0.03	0.13	111
Crypt depth, μm	145	139	144	138	147	140	145	0.45	0.04	0.23	0.70	0.29	0.79	0.31	18,5
Villi / Crypt ratio	7.49	7.06	6.96	6.67	7.67	7.06	7.28	<0.001	0.16	0.37	0.70	0.37	0.20	0.68	1,00
Goblet cells, n/300 μm	21.02	21.67	22.25	22.84	20.45	22.20	21.09	0.24	0.001	0.06	0.34	0.12	0.43	0.27	2.46
CD3^+^ cells, n/10,000 μm^2^	2242	2219	2297	2069	2436	2223	2283	0.16	<0.001	0.09	0.23	<0.001	<0.001	0.46	459
CD45^+^ cells, n/10,000 μm^2^	2879^ab^	2793^a^	2925^b^	2437	3295	2915	2816	0.02	<0.001	0.01	0.18	<0.001	0.01	0.45	537

*MSE* root mean square error. *C*, control diet. *CN*, control diet supplemented with 0.2% chestnut wood extracts. *GP*, control diet supplemented with 0.2% grape pomace extracts

^1^Averages of traits according to Age × Sex are provided in Table S1. ^2^Averages of traits according to Diet × Sex are provided in Table S2.

^a,b^Values with different superscript letters significantly differ *(P* < 0.05)

As the age increased from 15 to 35 d, villi height (*P* < 0.01), crypt depth (*P* < 0.05), and density of both CD3^+^ and CD45^+^ cells (*P* < 0.001) increased (Table 5), whereas the density of goblet cells decreased (*P* = 0.001).

Regarding sex, villi height was lower in females than in males (*P* < 0.01), whereas the densities of CD45^+^ cells (*P* = 0.01) and goblet cells (*P* = 0.06) were higher (Table 5). As for villi height, CD3^+^ and CD45^+^ cells, significant interactions were recorded between sex and age (Table S1), and between sex and diet (Table S2).

### Whole transcriptome analysis

**Fig. 1:**
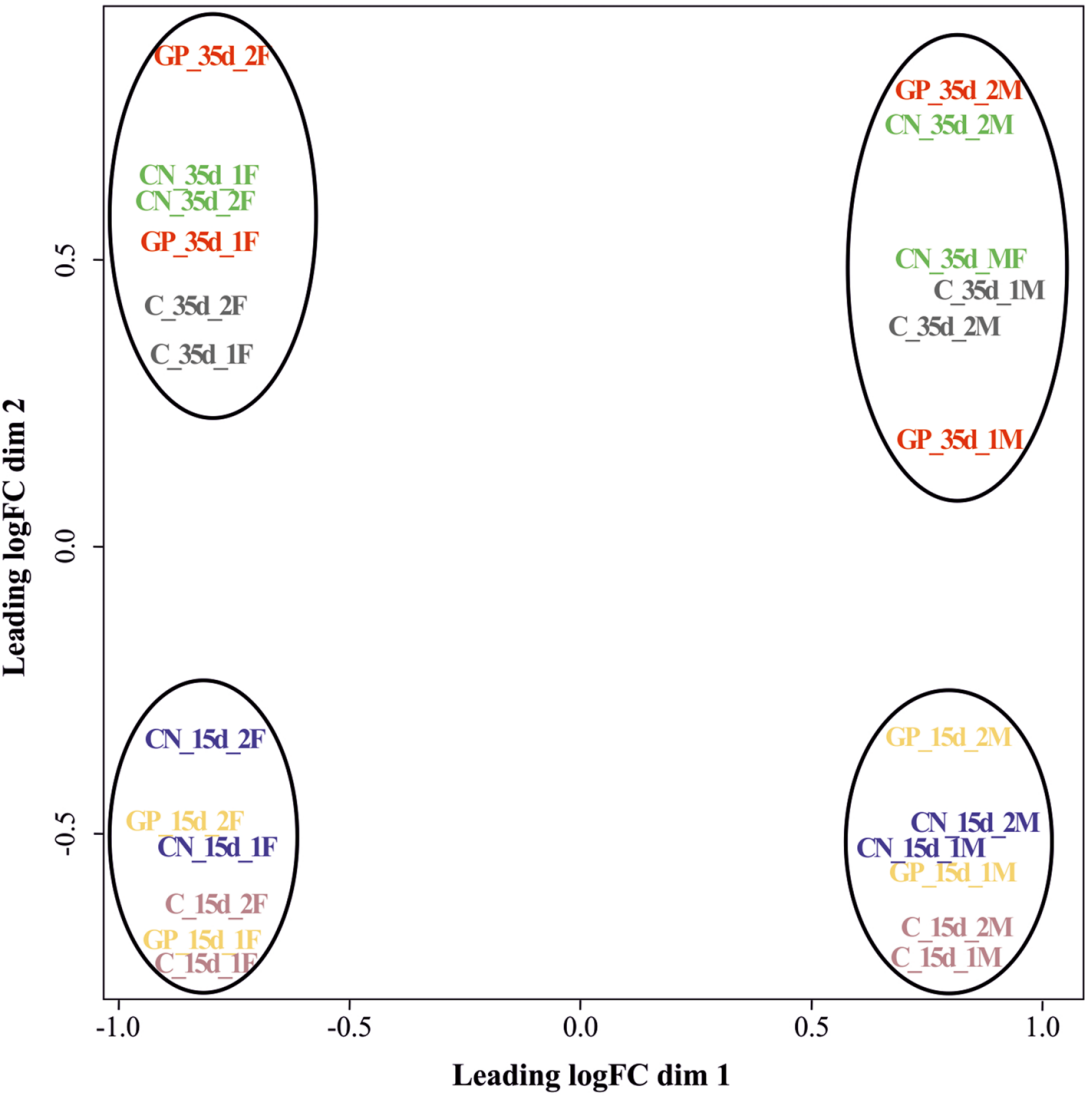
Multiple dimensional scaling (MDS) plot of distances between expression profiles (as log_2_ fold change, logFC) of RNA-seq libraries (24 samples) of the jejunum of female (F) and male (M) broiler chickens fed control diet (C diet), diet added with 0.2% chestnut extracts (CN diet), and diet added with 0.2% grape pomace extracts (GP diet) at 15 d (libraries C_15d, CN_15d, GP_15d) and 35 days of age (libraries C_35d, CN_35d, GP_35d) [1F, 2F, 1M, 2M stand for the two replicates for females and the two replicates for males per age]

A total of 725,278,263 raw reads were obtained. Raw Illumina sequencing data were deposited in GenBank under the BioProject accession number PRJNA666129. All samples passed the quality control measures. After trimming and rRNA removal, an average of approximately 30 million reads per sample was retained, with ~94% reads mapped to the chicken reference genome (Table S3). The multidimensional scaling plot provided unsupervised clustering of the samples (Fig. 1). The first dimension (*x*-axis) clearly separates females from males, whereas the second dimension (*y*-axis) separates samples by age. The biological variability within dietary treatments was low, as demonstrated by the clusters formed when MDS distances between expression profiles of all the replicates were plotted. Jejunum samples showed largely different transcriptional profiles between sexes and between ages, whereas compared with the C diet, the CN and GP diets had a moderate impact on jejunum gene expression.

### Dietary effects on jejunum whole transcriptome

Transcriptional profiles of same-sex and same-age chickens fed CN and GP diets were compared to those of chickens fed the C diet, thus identifying significant DEGs, that are shown in Table 6 and in the supplementary Tables S4 and S5.

**Table 6:** Number of significantly upregulated and downregulated differentially expressed genes (Fold change ≥ 2; False Discovery Rate ≤ 0.05) in the jejunum of male or female broiler chickens fed the experimental diets at 15 or 35 days of age

Comparison	Number of up-regulated genes	Number of down-regulated genes	Total DEGs
Female chickens at 15 d			
CN diet vs. C diet	7	8	15
GP diet vs. C diet	14	18	32
Male chickens at 15 d			
CN diet vs. C diet	7	7	14
GP diet vs. C diet	21	10	31
Female chickens at 35 d			
CN diet vs. C diet	18	12	30
GP diet vs. C diet	197	74	271
Male chickens at 35 d			
CN diet vs. C diet	8	8	16
GP diet vs. C diet	2	4	6

*DEGs* differentially expressed genes. *C*, control diet. *CN*, control diet supplemented with 0.2% chestnut wood extracts. *GP*, control diet supplemented with 0.2% grape pomace extracts

At 15 d, when comparing females fed the CN diet with those fed the C diet, 15 DEGs were identified. Cytochrome P450 1A1 (*CYP1A1;* logFC: 1.71), *CYP1A2* (logFC: 1.47) and STEAP4 Metalloreductase (*STEAP4*; logFC: 4.65) were upregulated. Guanylate-binding protein 1-like (*GBP1-like*; logFC: -8.43), butyrophilin subfamily 1 member A1-like (*BTN1A1-like*; logFC: -1.69), and leukocyte immunoglobulin-like receptor subfamily A member 2 (logFC: -4.12) were downregulated. Regarding the effects of the GP diet compared with those of the C diet in females at 15 d, avian beta-defensin 9 and 10 (*AvBD9* and *AvBD10*, respectively) and class I histocompatibility antigen, F10 alpha chain-like (*HA1F-like*) were remarkably upregulated (logFC of 7.18, 9.23, and 2.43, respectively). Both *GBP1*-*like* and *BTN1A1*-*like* mRNA levels were lowered (logFC: -6.09 and -1.45). Gap junction protein beta 1 and pyruvate dehydrogenase kinase 4 were among the most significantly downregulated genes.

At 35 d, 30 DEGs were detected when comparing females fed the CN and C diets. Some genes involved in immunity and inflammation, such as complement C1q C chain (logFC: 1.07), major histocompatibility complex class I polypeptide-related sequence A (*MICA*; logFC: 1.72), extracellular fatty acid-binding protein (logFC: 1.32), and bradykinin receptor B1 (*BDKRB1*; logFC: 1.26), were upregulated. Likewise, the glutathione *S*-transferase class-alpha (*GSTA*) gene was upregulated (logFC: 1.25).

In females at 35 d, compared with the C diet the GP diet upregulated 197 genes. Among the genes involved in inflammatory processes, the following were upregulated: nephroblastoma overexpressed (*NOV*; logFC: 1.06), netrin 1 (logFC: 1.05), C1q and tumor necrosis factor related protein 4 (*C1QTNF4*; logFC: 1.69), and *BDKRB1* (logFC: 1.09). Additional upregulated genes that play a role in the regulation of immune defense were the junctional adhesion molecule 2 (logFC: 1.04), C-X-C motif chemokine ligand 12 (*CXCL12*; logFC: 1.08), and T-cell surface glycoprotein CD8 alpha chain-like (logFC: 1.74). Moreover, the mRNA levels of hemoglobin beta, subunits A, A1, and AD (*HBBA*, *HBA1*, and *HBAD*), as well as *GSTA*, were increased (logFC: 2.25, 1.85, 2.27, and 1.18, respectively). Finally, as for upregulated genes, glucagon like peptide 1 receptor (*GLP1R*; logFC: 1.02), and solute carrier family 2, facilitated glucose transporter member 4-like (*GLUT4-like*; logFC: 1.17) play a role in nutrients intestinal absorption. Among the downregulated genes, some are involved in vitamin and protein absorption, such as scavenger receptor class B member 1 (logFC: -2.00) and beta-carotene oxygenase 1 (logFC: -1.55). Further downregulated genes are involved in protein and carbohydrate metabolism, such as carboxypeptidase O (logFC: -1.80), glutamic-pyruvic transaminase 2 (logFC: -1.27), and lactase (logFC: -1.10). Additionally, the expression of *AvBD9* and *AvBD10* was downregulated (logFC: -7.59 and -6.21, respectively).

At 15 d, compared with the C diet, the CN diet significantly upregulated *STEAP4* (logFC: 3.31), *CYP1A* (logFC: 1.89), and *HA1F-like* (logFC: 1.61) genes in males. Among the seven downregulated genes, ectonucleotide pyrophosphatase/phosphodiesterase 7 (logFC: 1.66) encodes a protein that protects the intestinal mucosa from inflammation. The gene most significantly induced by the GP diet was glutathione peroxidase 4 (logFC: 1.26), whereas the suppressor of cytokine signaling 3 (logFC: -1.04) and *IL-22* (logFC: -2.80) were downregulated.

At 35 d, CN diet in males significantly induced *HA1F-like* (logFC: 1.46) and *MICA* mRNA levels (logFC: 1.41), while lysozyme-g-*like* (logFC: -2.47) and *NOV* (logFC: -1.89) were downregulated.

**Fig. 2:**
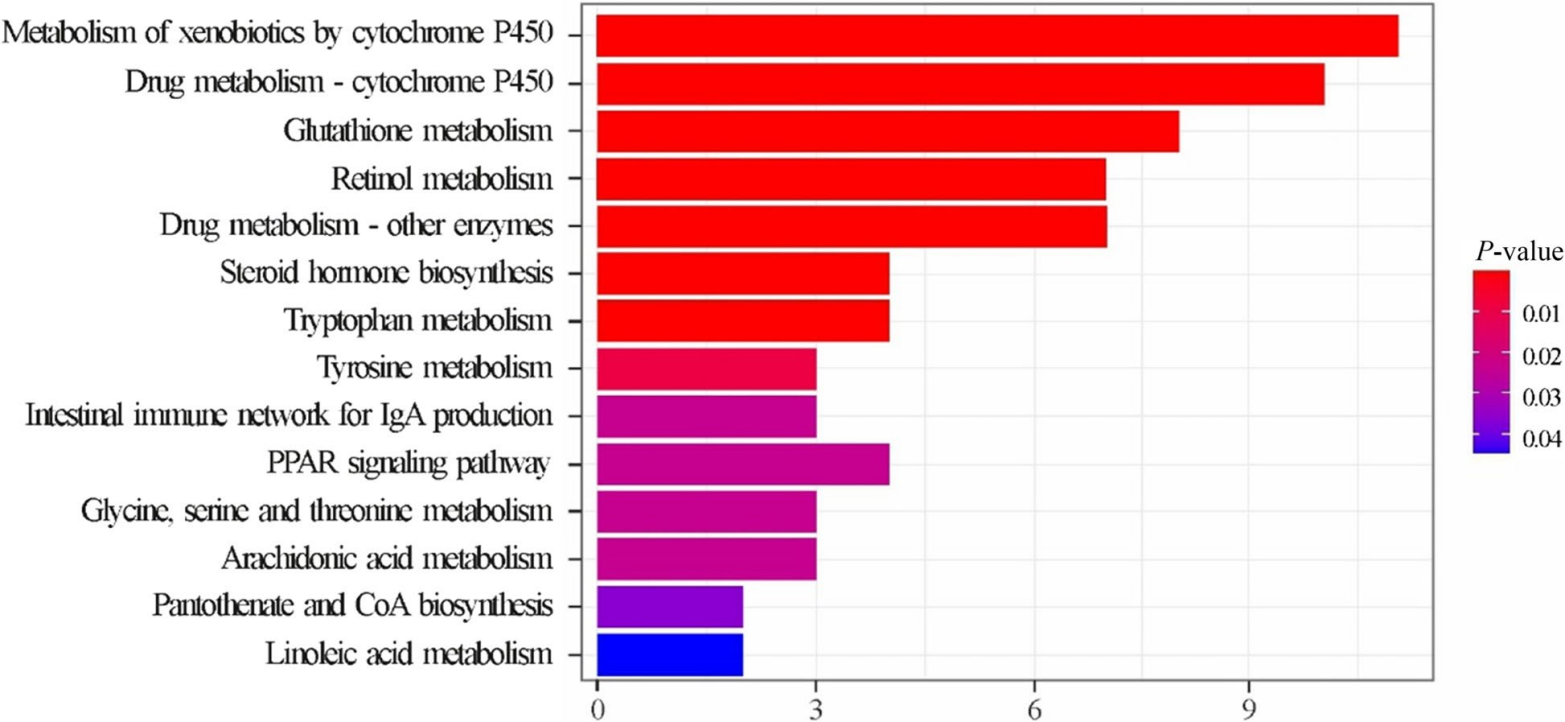
Over-represented KEGG pathways among the upregulated genes in broiler chickens at 35 d compared with those in broiler chickens at 15 d. The number of differentially expressed genes (DEGs) belonging to each enriched KEGG pathway is reported in the x axes. Color gradient represents the *P*-value significance, as specified in the legend. *P*-values were adjusted using the Benjamini–Hochberg method

On comparing chickens at 35 d and those at 15 d (Table S6), 324 DEGs were identified (FDR ≤ 0.05; FC ≥ 1.5). The top 10 upregulated and downregulated genes are shown in Table 7. Only two KEGG pathways were statistically enriched by the 213 genes upregulated after 15 d, namely, the “peroxisome proliferator-activated receptor (PPAR) signaling pathway” and the “neuroactive ligand–receptor interaction pathway” (Table S7). However, a higher number of KEGG pathways (i.e., 14) were significantly enriched by the upregulated genes in chickens at 35 d (Fig. 2 and Table S7); some of them were related to PPAR signaling, xenobiotic metabolism, glutathione metabolism, steroid hormone synthesis, IgA production, and amino acid metabolism.

**Table 7:**
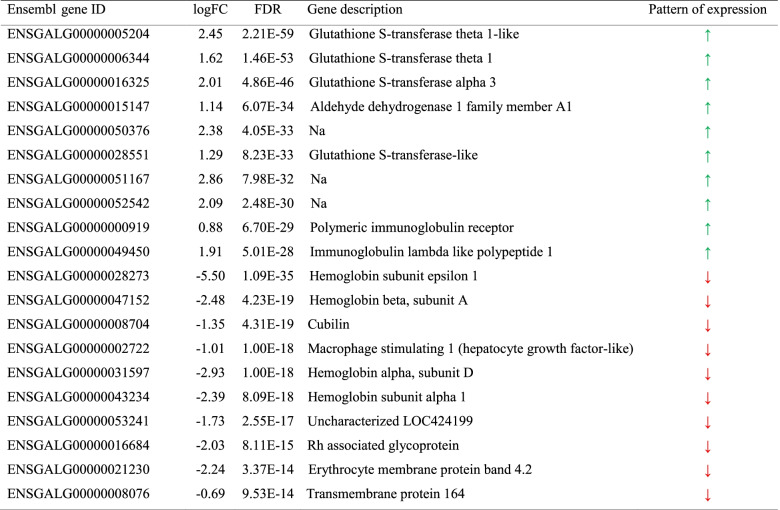
Top 10 upregulated (  ) and downregulated (  ) differentially expressed genes (DEGs) in broiler chickens at 35 d compared with those at 15 d. For each DEG, Ensembl gene ID, log_2_ fold change (logFC), and False Discovery Rate (FDR), as reported in edgeR output and the Ensembl gene description, are provided

**Fig. 3:**
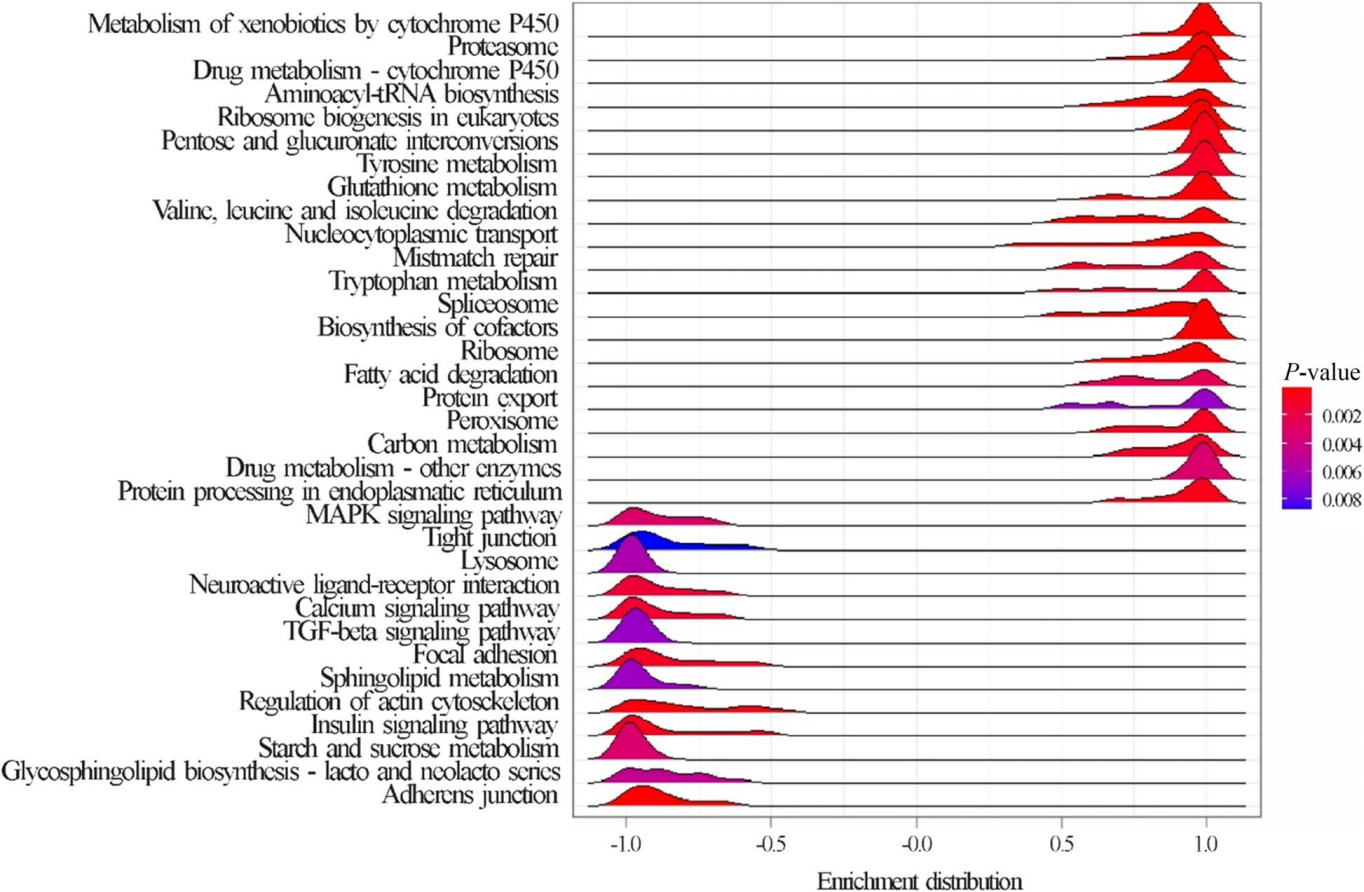
Results of the Gene Set Enrichment Analysis (GSEA): top-35 significantly enriched KEGG pathways in broiler chickens at 35 d compared with those in broiler chickens at 15 d. Pathways showing an enrichment value > 0 are activated in 35-d broiler chickens, whereas those with an enrichment value < 0 are activated in 15-d broiler chickens. Color gradient represents the *P*-value significance, as specified in the legend. The *P*-values were adjusted using the Benjamini–Hochberg method

In 15-d chickens, the GSEA (Table S7) pointed out the activation of 18 KEGG pathways related to cell–cell junctions (i.e., tight junction, focal adhesion, and adherent junction), cytoskeleton, and transforming growth factor-β (TGF-β). In 35-d chickens, the GSEA highlighted the activation of 33 KEGG pathways; the most significant ones include gene sets related to xenobiotic metabolism, antioxidant response (i.e., glutathione metabolism), protein processing (i.e., proteasome, protein export, and ribosome), and amino acid metabolism. The top-35 significant KEGG pathways activated in 35-d chickens compared to 15-d chickens are reported in Fig. 3.

**Fig. 4:**
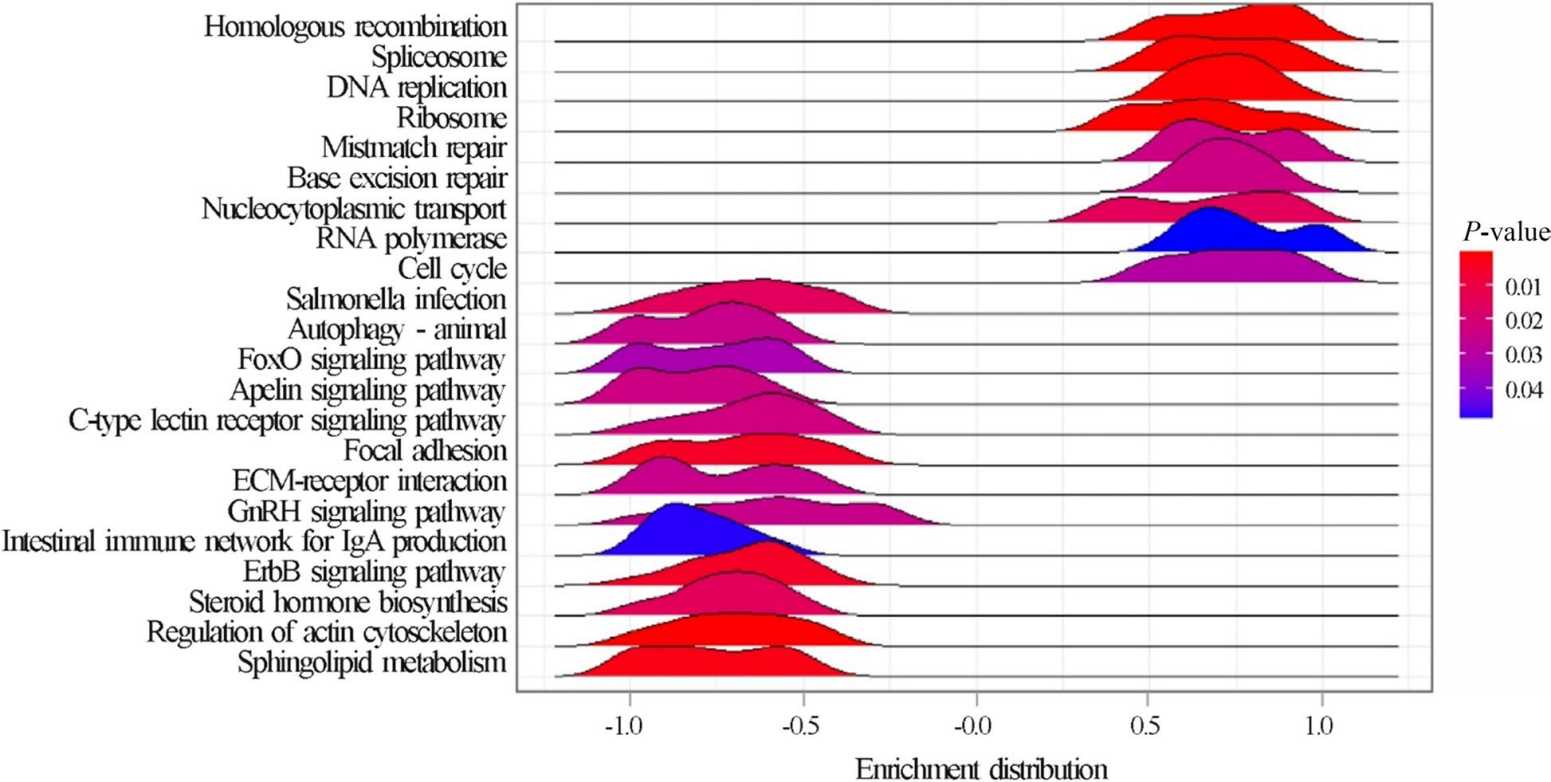
Results of the Gene Set Enrichment Analysis (GSEA): enriched KEGG pathways in female broiler chickens compared with those in male broiler chickens. Pathways showing an enrichment value > 0 are activated in females, whereas those with an enrichment value < 0 are activated in males. Color gradient represents the *P*-value significance, as specified in the legend. The *P*-values were adjusted using the Benjamini–Hochberg method

On comparing females and males, 246 significant DEGs were identified; among these, 47 and 199 genes were upregulated in females and males, respectively. The top 10 upregulated and downregulated genes are listed in Table 8. The KEGG enrichment analysis of upregulated genes in males showed that only the “ribosome biogenesis in eukaryotes” pathway was significantly enriched. In contrast, GSEA identified a total of 19 KEGG pathways that were significantly enriched, 9 in females and 13 in males (Fig. 4; Table S7). In females, a considerable number of activated gene sets was related to cell cycle regulation (e.g., DNA replication and mismatch repair).

**Table 8:**
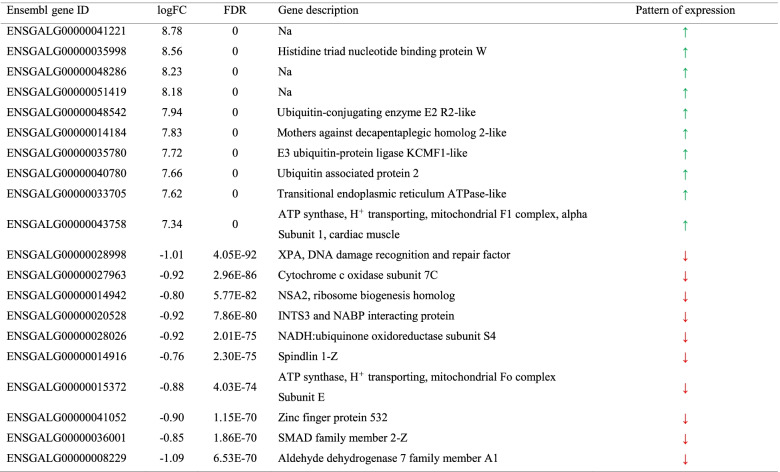
Top 10 upregulated (  ) and downregulated (  ) differentially expressed genes (DEGs) in females compared with those in males. For each DEG, Ensembl gene ID, log_2_ fold change (logFC), and False Discovery Rate (FDR), as reported in edgeR output and the Ensembl gene description, are provided

## Discussion

In animal feeding, tannins can have either beneficial or detrimental effects depending on their chemical structure and dosage, in addition to other individual (e.g., animal species, age, sex, physiological state) and feeding factors [43]. Standardization of conditions (in terms of quantity and quality of the different polyphenolic fractions) is even more difficult when using extracts from natural sources. Thus, in the present trial, we compared two products of a similar nature (byproducts containing different quantities of polyphenols of different types) in the same form (powder) at the same moderate supplementation level.

We observed that the GP diet improved growth performance compared with the other dietary treatments, whereas CN diet reduced the villi height compared to the control diet and the density of CD45^+^ cells compared to the GP diet. The highest growth performance measured in broilers fed GP diet might be in part supported by an increased intestinal absorption of nutrients. In the present study, this is suggested by the upregulation in females of genes such as the receptor of glucagon-like peptide (*GLP1R*), that promotes efficient nutrient assimilation [44], and *GLUT4*, a glucose transporter whose expression can be induced by tannins [45].

The different nature of tannins of CN and GP and their effects at the gut level can partly explain our results, even if different effects are likely to expected depending on the animal age and gut tract [46]. The CN mainly contains hydrolysable tannins, which usually possess a relatively low molecular weight and high bioavailability [8, 9, 47]. Thus, CN hydrolysable tannins can be early hydrolyzed and absorbed in the first gastro intestinal tract, as observed in rats [48], exerting both cytotoxic or cytoprotective effects on jejunal mucosa [46, 48, 49]. On the other hand, GP condensed tannins can remain active along the whole gut and can be transformed by gut microbiota in other bioactive metabolites which can further affect gut mucosa and microbiota composition [46, 50].

Schiavone et al. [8] showed dose-dependent effects of dietary supplementation with CN tannins, i.e., null at the lowest inclusion rate (1.5 g CN wood extract/kg), positive at the intermediate level (2.0 g/kg) in terms of final live weight and feed intake, and negative at the highest rate (2.5 g/kg), which reduced live weight, compared with the C diet (from 14 to 56 days of age). Similarly, Jamroz et al. [10] did not observe effects on performance with low CN tannin inclusion rates (0.25 and 0.50 g of sweet CN tannin/kg), whereas the highest dose (1.0 g/kg) decreased the live weight at 41 d and caused alterations in the intestinal wall morphology and a decreased proliferation rate in the mother-cell zone. These findings are consistent with the decreased villi length observed in our study in chickens fed the CN diet. In contrast, CN tannin supplementation increased the average daily weight gain and jejunum villus height under a heat-stress challenge [51] and reduced the proliferation of *Clostridium perfringens* and the severity of gut damage in necrotic enteritis [52].

Similar to CN, condensed tannins of GP byproducts (extracted from seeds, skin, and stems as byproducts of winemaking) can reduce diet digestibility and negatively affect the growth performance of monogastric animals [53]. In poultry, during the first period (from hatching until 21 days of age), the increase in GP supplementation (from 2.5 to 5.0 g of GP seed extract/kg) decreased both animal performance and diet digestibility [54]. Some authors reported negative effects on performance with high inclusion rates of grape-based extracts [12], which also showed a negative effect on villus length. In contrast, 6% dietary inclusion of a GP concentrate did not modify performance or diet digestibility but showed antioxidant potential that was as effective as vitamin E in diet, excreta, ileal content, and breast muscle [15]. Other authors evidenced that the dietary supplementation of grape byproducts increased activity of total superoxide dismutase and decreased the content of malondialdehyde in plasma [19] and leg meat [55], whereas this was not observed in the present study when measuring meat TBARs.

In our study, we neither observed any effect of CN or GP supplementation on myopathy rate, which is consistent with the findings of previous studies that tested various antioxidants for this purpose [56–58]. Nevertheless, oxidative stress, localized hypoxia, increased intracellular calcium, and the presence of muscle fiber-type switching are pathways responsible for the occurrence of WB and WS [16, 59, 60]; hence, the administration of antioxidant substances, such as GP or CN extracts, could be expected to affect myopathy occurrence.

In the present study, the increased mucosal immune responses with the GP diet (i.e. the higher density of intraepithelial leukocytes, especially CD45^+^) compared to the CN diet could be attributed to the tannin nature and availability along the intestinal tract as discussed above (condensed compared to hydrolysable tannins in GP compared to CN). On the other hand, when comparing different dietary supplementation doses of tannic acid, effects on broiler chicken immunity changed from positive to negative in a dose-dependent manner [61]. Indeed, genes involved in either pro- or anti-inflammatory response pathways and antimicrobial responses were affected by GP and CN diets, besides genes contributing to the antioxidant responses. Nevertheless, the majority of these genes, especially those related to immune functions, were significantly regulated by the GP supplementation consistently with the higher CD45^+^ density in the jejunum of broilers fed GP diet compared to those fed CN diet. Finally, the improved immune competences observed in broilers fed GP diet might have positively impacted on their growth performance compared to the other dietary treatments.

In the present study, the majority of changes in the chicken jejunum transcriptome was observed in chickens of the same sex but at different ages. In fact, in 15-d females, the GP and CN diets downregulated genes involved in the inflammatory response [62] and protection against microbes and viruses, such as *GBP1-like* [63] a robust marker of inflammation*.* In 15-d male and female broilers, the CN diet upregulated *STEAP4*, which plays a role in the response to chronic inflammation in colon cancer [64], the suppression or inhibition of cytokine production and signaling (IL-6 and TNF-α-induced NF-κB signaling [65]), the response to nutrients, oxidative stress, fatty acid metabolism, and glucose metabolism [66]. In 15-d chickens of both sexes, CN supplementation modulated the expression of *CYP1A*, an important detoxifying monooxygenase that can be induced by natural polyphenols [67] as demonstrated in the gut of pigs fed CN extracts [68].

In 35-d females, the GP diet upregulated *C1QTNF4* (involved in the regulation of the inflammatory networks and in feed intake suppression in mice) [69] and *CXCL12* (a constitutive and inflammatory chemokine of the intestinal immune system) [70]. Conversely, in 35-d females, the GP diet downregulated beta-defensins (*AvBD9*, *AvBD10*), which possess modest antimicrobial properties and display a wide range of immunomodulatory activities, such as modulation of pro- and anti-inflammatory responses, promotion of wound healing [71]. Additionally, in 35-d females, both GP and CN diets induced the expression of *GSTA*, primarily involved in the defense against oxidative stress. The GP diet also induced three hemoglobin subunits (i.e., *HBBA*, *HBAD*, and *HBA1*), which are upregulated in the presence of oxidative stress and are believed to alleviate it [72].

Notably, most of the differences in favor of GP supplementation regarding the overall transcriptomic response appeared in the second period of growth. Overall, this is consistent with the results of Farahat et al. [73] and Yang et al. [19], who observed a time-dependent cumulative effect of dietary supplementation with grape seed extracts and pomace concentrate. However, according to some authors [8, 53], the dietary supplementation with tannins (from grape seed and CN extracts) was more effective in younger broilers than in older ones, likely based on their effects on pathogenic microorganisms as well as on commensal microbiota [74, 75].

In the present study, the most significant age-dependent transcriptional variations (i.e., top 10 upregulated DEGs) were related to immunoglobulin functions, for which the KEGG pathway “intestinal immune network for IgA production” was significantly enriched with the increase in age. Overall, this age-dependent improvement of immune competencies, together with the increased densities of jejunum CD3^+^ and CD45^+^ cells we observed in older chickens, was expected. Additionally, four of the top 10 upregulated DEGs encoded GSTs, which suggests that glutathione-dependent detoxifying capability significantly increases with chicken development. This was further strengthened by the enrichment of the KEGG pathways “glutathione metabolism” and “metabolism of xenobiotics by cytochrome P450,” both including several differentially regulated *GST*s.

The downregulation of three hemoglobin subunits (i.e., *HBBA*, *HBAD*, and *HBA1*) in 35-d chickens compared with 15-d chickens could be related to an imbalance between the muscular development of birds and the vascularization/blood supply and might reflect the possible muscle suffering that leads to muscle fiber degeneration and myopathies with the increase in age [76, 77].

Expected differences in performance between males and females were confirmed and found to be associated with differences in the jejunum transcriptome. Top upregulated genes in females are involved in ubiquitination, a post-translational mechanism for protein degradation via the proteasome, ensuring the structural integrity control and/or protein turnover rate. However, these transcriptional variations seem related to DNA repair pathways and cell fate decisions, rather than proteolytic processes. This hypothesis is consistent with a non-degrading role of protein ubiquitination [78], and supported by the enriched KEGG pathways reported in females (e.g., “mismatch repair,” “cell cycle”).

Overall, the gene expression data showed that DNA repair processes are likely to be differentially regulated in male and female jejunum. Notably, sex differences in the control of cell cycle and DNA repair have been reported in mammals [79, 80]. Upregulated DEGs playing a role in the mitochondrial respiratory chain and ATP production were also reported, e.g., cytochrome c oxidase subunit 7C and NADH:ubiquinone oxidoreductase subunit S4. This is most likely linked to the different growth rate between sexes (higher in males), which is confirmed by the significant activation in males of genes involved in tissue morphogenesis and maintenance of cell and tissue structure and function (e.g. “focal adhesion,” “ECM-receptor interaction,” and “regulation of actin cytoskeleton” gene sets). On the other hand, differences in growth rates and final live weight between sexes are also associated to a different occurrence of myopathies whereas the results of the present study confirmed that SM occurred more often in females than in males, whereas WB was more frequent in males than in females [81, 82].

## Conclusions

The dietary supplementation of GP extracts can be beneficial to broilers because it increases growth performance and the final live weight of animals. In the absence of any specific challenges, it still improved the jejunum morphology and the overall immune response. The addition of CN extracts affected the jejunum morphology, although growth traits remained unaffected. Overall, the chicken jejunum transcriptome was scarcely affected by extracts, although in 35-d females fed the GP diet, some positive effects on nutrient absorption, immune and antioxidant responses were observed. Regarding age and sex, further molecular investigations are required in view of the interactions with dietary additives, as observed in the present study, and subsequent effect on chicken health and performance.

## Supplementary Information

**Additional file 1.** RNA-seq libraries sequenced and transcriptional changes. Averages of traits for jejunum morphology and immuno-histochemical analyses according to interactions between sex and age and between sex and diet are provided in Table S1 and Table S2, respectively. Details on sequenced RNA-seq libraries are provided in Table S3. Comparisons among transcriptional profiles of same-sex and same-age chickens fed CN and GP diets compared to those of chickens fed the C diet, thus identifying significant DEGs, are shown in supplementary Tables S4, S5 and S6. Annotated genes significantly regulated in more than one comparison are listed in Tables S5. Gender-related and sex-related transcriptional changes are reported in Tables S5 and S6. Functional enrichment analysis is shown in Tables S7.

## Data Availability

Raw Illumina sequencing data have been deposited in GenBank (SRA) under the SRA accessions SRR12728661-SRR12728684, and they will be published after manuscript acceptance. During reviewing process, BioProject’s metadata are available at the following link: https://dataview.ncbi.nlm.nih.gov/object/PRJNA666129?reviewer=398l4cbdnfnmu1mris3g20ol1c The other datasets analysed in the current study are available from the corresponding author upon reasonable request.

## Abbreviations

ANOVA: Analysis of variance
AvBD10: Avian beta-defensin 10
AvBD9: Avian beta-defensin 9
BDKRB1: Bradykinin receptor B1
BTN1A1: Butyrophilin subfamily 1 member A1
C: Control
C1QTNF4: C1q and tumor necrosis factor related protein 4
CN: Chestnut wood
GP: Grape pomace
CXCL12: C-X-C motif chemokine ligand 12
CYP1A1: Cytochrome P450 1A1
DE: Differential expression
DEGs: Differentially expressed genes
FC: Fold change
FDR: False discovery rate
GBP1: Guanylate-binding protein 1
GSEA: Gene Set Enrichment Analysis
GSTA: Glutathione S-transferase class-alpha
HA1F-like: F10 alpha chain-like
HBA1: Hemoglobin beta subunit A1
HBAD: Hemoglobin beta subunit AD
HBBA: Hemoglobin beta subunit A
IL: Interleukin
MICA: major histocompatibility complex class I polypeptide-related sequence A
NOV: Nephroblastoma overexpressed
PAS: Periodic Acid-Schiff
PBS: Phosphate-buffered saline
PPAR: Peroxisome proliferator-activated receptor
pval: *P* values
rRNAs: Ribosomal RNAs
SM: Spaghetti meat
STEAP4: Metalloreductase
TBARs: Thiobarbituric acid reactive substances
TGF-β: Transforming growth factor-β
TNF-α: Tumor necrosis factor Alpha
WB: Wooden breast
WS: White striping
